# Right minithoracotomy and resternotomy approach in patients undergoing a redo mitral valve procedure

**DOI:** 10.1093/icvts/ivab228

**Published:** 2021-08-15

**Authors:** Nadejda Monsefi, Basel Makkawi, Mahmut Öztürk, Hossien Alirezai, Eissa Alaj, Farhad Bakhtiary

**Affiliations:** 1 Department of Cardiac Surgery, University Hospital Bonn, Bonn, Germany; 2 Department of Cardiac Surgery, Helios Heart Center Siegburg, Siegburg, Germany

**Keywords:** Mitral valve, Minimally invasive surgery, Sternotomy, Video-assisted redo valve procedures

## Abstract

**OBJECTIVES:**

A minimally invasive approach via a thoracotomy is an alternative in challenging redo cardiac procedures. Our goal was to present our early postoperative experience with minimally invasive cardiac surgery via a right minithoracotomy (minimally invasive) and resternotomy in patients undergoing a mitral valve procedure as a reoperation.

**METHODS:**

From 2017 until 2020, reoperation of the mitral valve was performed through a right-sided minithoracotomy in 27 patients and via a resternotomy in 26 patients. Patients with femoral vessels suitable for cannulation underwent a minimally invasive technique. Patients requiring concomitant procedures regarding the aortic valve were operated on via a resternotomy.

**RESULTS:**

The mean age was 66 ± 12 years in the minimally invasive group and 65 ± 12 years in the whole cohort. The average Society of Thoracic Surgeons score was 11 ± 10% in the minimally invasive group and 13 ± 9% in all patients. The majority of the patients underwent reoperation because of severe mitral valve insufficiency (48% and 55%, respectively). The mean time to reoperation was 7 ± 9 years (minimally invasive group). The 30-day mortality was 4% in the minimally invasive group and 11% in the whole cohort. The blood loss was 566 ± 359 ml in the minimally invasive group and 793 ± 410 ml totally. There were no postoperative neurological complications in the minimally invasive group and 1 (2%) in the whole cohort. Postoperative echocardiography revealed competent mitral valve/prosthesis function in all patients.

**CONCLUSIONS:**

A minimally invasive approach for a mitral valve reoperation in selected patients is a safe alternative to resternotomy with a low transfusion requirement. Both surgical techniques are associated with good postoperative outcomes.

## INTRODUCTION

The adverse events during redo cardiac surgery procedures, especially in repeat sternotomy, are well known and lead to higher operative risk, postoperative morbidity and mortality [1, 2]. The repeat sternotomy approach is challenging because of severe adhesions, complex valve exposure and an increased risk of injury to cardiac structures [3]. Mitral valve procedures, even in redo cardiac surgery, can also be performed with a minimally invasive technique through a right-sided thoracotomy. Carpentier *et al.* reported the minimally invasive technique for mitral valve procedures using a video-assisted right minithoracotomy in 1996, and many other international cardiac surgery centres have adopted this innovative technique over time [4–8]. The minimally invasive technique is a major advance in cardiac surgery in terms of innovation and postoperative morbidity. The evolution of this surgical approach offered the ability to avoid the need for a resternotomy and injuries related to re-entry. Furthermore, the idea behind the minimally invasive technique was to reduce blood loss and postoperative morbidity [9, 10]. However, only a few studies have been published about redo minimally invasive mitral valve procedures, and there are no randomized trials investigating the safety and efficacy of these procedures [11–13]. The goal of our study was to present our early postoperative results with the minimally invasive and the resternotomy approaches in patients undergoing a mitral valve procedure as a reoperation.

## MATERIALS AND METHODS

### Study design

From 2017 until 2020, a total of 53 patients underwent a reoperation of the mitral valve at our centre, either via a right minithoracotomy (minimally invasive, *n* = 27), or a resternotomy (*n* = 26). During the same period, 781 mitral valve procedures were performed.

### Indication for mitral valve surgery

The indication for a mitral valve procedure was mixed mitral valve disease in 48% (*n* = 13), severe mitral valve regurgitation (MR) in 48% (*n* = 13) and endocarditis in 4% (*n* = 1) in the minimally invasive group. We defined mixed mitral valve disease as a combination of mitral valve insufficiency and stenosis. The patients with this pathology had severe mitral valve insufficiency and calcified leaflets with moderate stenosis of the mitral valve. Patient preoperative characteristics are presented in Table 1. This retrospective study was approved by the local ethics committee.

**Table 1:** Preoperative patient demographics

Baseline characteristics	Minimally invasive procedure	Resternotomy and minimally invasive procedure
Number of patients	27	53
Age (years), mean ± SD	66 ± 12	65 ± 12
Systemic hypertension, *n* (%)	15 (56)	34 (64)
Male gender, *n* (%)	13 (48)	25 (47)
Mitral valve endocarditis, *n* (%)	1 (4)	5 (9)
Mixed mitral valve disease, *n* (%)	13 (48)	19 (36)
Severe mitral valve insufficiency, *n* (%)	13 (48)	29 (55)
Diabetes mellitus, *n* (%)	6 (22)	14 (26)
COPD, *n* (%)	3 (11)	6 (11)
Chronic renal failure, *n* (%)	6 (22)	15 (28)
CAD involvement, *n* (%)	6 (22)	18 (34)
STS score (%), mean ± SD	11 ± 10	13 ± 9
EuroSCORE II (%), mean ± SD	7 ± 3	9 ± 6
NYHA class, mean ± SD	3 ± 0.5	3 ± 0.5
LVEF (%), mean ± SD	53 ± 9	50 ± 14
LVEDD (cm), mean ± SD	54 ± 11	55 ± 10
Previous cardiac surgery, *n* (%)		
Mitral valve repair	18 (66)	22 (42)
Mitral valve replacement	1 (4)	5 (9)
CABG	2 (7)	13 (25)
Tricuspid valve repair	1 (4)	2 (4)
Aortic valve replacement	5 (19)	11 (21)
ASD correction	0	1 (2)

ASD: atrial septal defect; CABG: coronary artery bypass grafting; CAD: coronary artery disease; COPD: chronic obstructive pulmonary disease; LVEDD: left ventricular end-diastolic diameter; LVEF: left ventricular ejection fraction; NYHA: New York Heart Association; SD: standard deviation; STS: Society of Thoracic Surgeons.

### Inclusion criteria for a minimally invasive procedure

Patients requiring reoperative mitral valve surgery who had femoral vessels suitable for cannulation were included in the minimally invasive group. Routine ultrasound examinations and computed tomography scans were performed on admission to measure the diameter of the femoral artery. Furthermore, a preoperative angiogram was done to rule out coronary artery disease (CAD).

### Inclusion criteria for a resternotomy approach (exclusion criteria for a minimally invasive procedure)

A diameter of <7 mm for the common femoral artery was an exclusion criterion for the minimally invasive approach. These patients underwent resternotomy. Patients with peripheral artery disease, distinct kinking or a history of type B aortic dissection were also operated on via a resternotomy.

In our experience, cannulation of small vessels with a diameter of <7 mm was feasible; however, the fragile wall of the vessel had to be reconstructed at the end of the operation due to local dissection or a tear in the tissue. We have made these observations about the minimally invasive mitral and aortic valve procedures previously. For safety reasons, we excluded patients with a femoral artery diameter of <7 mm from the minimally invasive procedure to minimize vascular complications.

### Echocardiography

Preoperative, intraoperative and postoperative echocardiographic examinations were performed routinely in all patients.

### Study end points

The primary end point was 30-day mortality. We defined the secondary end points as postoperative stroke, blood loss, a pacemaker implant, wound infection, vascular complications related to cannulation for the heart lung machine, ventilation time, in-hospital stay and reoperation on the mitral valve.

### Surgical procedure

#### Minimally invasive approach

**Figure 1:**
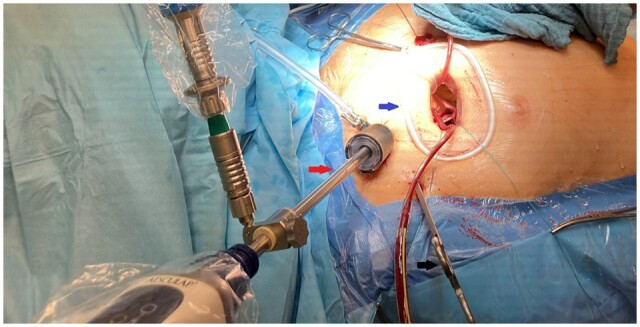
Minimally invasive mitral valve procedure via a video-assisted right minithoracotomy. Blue arrow: right minithoracotomy approach. Red arrow: port site for 3-dimensional videoscope. Black arrow: aortic clamp (Chitwood clamp).

**Figure 2:**
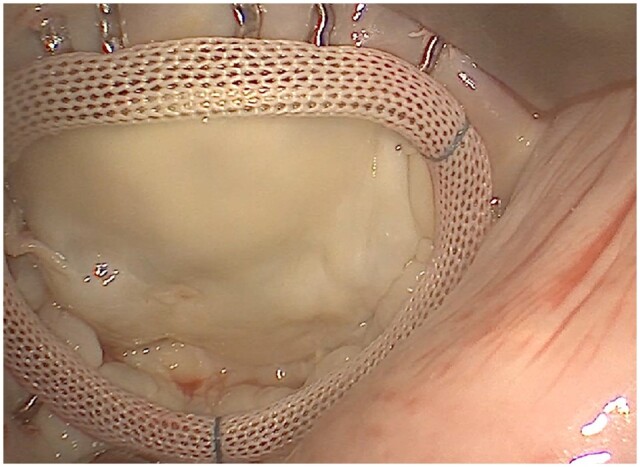
Visualization of mitral valve annuloplasty.

The patients were placed in a supine position with moderate elevation of the right side. We used the operative setting previously published by Seeburger *et al.* [13]. In detail, carbon dioxide was inserted in the situs to prevent an air embolism. The cannulation strategy for cardiopulmonary bypass (CPB) was femoro-femoral (usually 17–18 Fr × 15 cm arterial cannula and 21 or 25 Fr × 65 cm multiport venous drainage catheter, Edwards Lifesciences, Irvine, CA, USA). An additional right internal jugular vein cannula (18 Fr × 15 cm, FEMII018A, Edwards Lifesciences) was established by the anaesthesiologist for venous drainage if the patient’s weight was >80 kg or for a concomitant tricuspid valve procedure. A right minithoracotomy with a 4- to 7-cm incision in the fourth or fifth intercostal space was performed (Fig. 1). To obtain a better surgical exposure, a soft tissue retractor (Geister, Tuttlingen, Germany) and a rib retractor (Geister) were inserted. Port incisions were made for placing a videoscope (Aesculap, Tuttlingen, Germany) and a left atrial retractor. We used a 3-dimensional videoscope that was inserted via a port site (Fig. 1) to visualize the situs. Dense pleural and lung adhesions were carefully dissolved. After mobilizing the ascending aorta, a vent/cardioplegia catheter was placed into its ventral side. Then the ascending aorta was clamped with the Chitwood clamp through an additional small skin incision localized laterally in the second or third right intercostal space. The mitral valve procedure (Fig. 2) was performed using long-shafted instruments (Geister). An automatic suture device for the annular/subannular sutures (CorKnot, LSI solutions, Victor, NY, USA) was used routinely.

#### Resternotomy approach

We made a standard midline incision for the resternotomy. Dense pericardial adhesions were dissolved carefully. Usually, we performed central cannulation of the ascending aorta and the superior and inferior vena cava. In cases of dense adhesions of the ascending aorta, the right axillary artery was chosen for arterial cannulation. Carbon dioxide was inserted in the situs to prevent an air embolism. A vent/cardioplegia catheter was placed into the ascending aorta. The ascending aorta was cross-clamped. The mitral valve repair (MVR) or replacement technique was the same as that used in the minimally invasive group.

#### Mitral valve repair/replacement technique

The mitral valve was approached via the interatrial groove. Ruptured chordae were repaired with neochordae implants by applying the loop technique with pledget-armed Gore-Tex CV-5 sutures (W. L. Gore Inc., Newark, DE, USA). In addition, annuloplasty was used to stabilize the repaired valve or to treat the dilated mitral annulus (Fig. 2). Leaflet repair for cleft closure was performed with Cardionyl sutures (Peters Surgical US, Plymouth, MA, USA). Degenerated mitral valves were replaced after resection of the anterior leaflet and the corresponding chordae. The posterior leaflet was preserved to keep the annulopapillary continuity. Transoesophageal echocardiography was applied intraoperatively to evaluate mitral valve function after repair/replacement and for sufficient de-airing.

### Concomitant procedures

Concomitant tricuspid valve repair with annuloplasty and tricuspid valve replacement were performed after the mitral valve procedure. Surgical access was via the right atrium. We excluded the superior vena cava and the inferior vena cava with large ‘bulldog’ clamps or vessel loops to avoid accumulation of blood in the right atrium. In patients with additional aortic valve stenosis (resternotomy group), the aortic valve was replaced with a biological stented prosthesis through an aortotomy. In case of excessive endocarditis of the aortic root, the Bentall operation using a biological conduit was performed (resternotomy group).

### Myocardial preservation

The heart was arrested with cold crystalloid cardioplegia that was given antegrade via the aortic root or directly via the coronary ostia in case of significant aortic valve insufficiency. We used Bretschneider’s [14] histidine-tryptophane-ketogluterate solution (Custodiol, Köhler-Chemie, Alsbach-Hähnlein, Germany).

### Open left internal mammary artery/right internal mammary artery grafts

We had 2 patients who had previous coronary artery bypass graft (CABG) procedures before a minimally invasive mitral valve procedure. These 2 patients had open left internal mammary artery (*in situ*) to left anterior descending bypass grafts and saphenous vein (aorto-coronary) to right coronary artery and the ramus circumflex bypass grafts. We prepared the adhesions carefully. It was not possible to reach the left internal mammary artery graft via a minimally invasive approach. CPB was initiated, the aorta was clamped and cold crystalloid cardioplegia was applied antegrade via the aortic root. There were no cases of significant aortic valve insufficiency (≥grade 2). We used Bretschneider’s histidine-tryptophane-ketogluterate solution (Custodiol, Köhler-Chemie) and used hypothermia (28°C). The heart was under ventricular fibrillation during the procedure. The clamp time was 39 min, and 29 min in these 2 patients, respectively; the operative and postoperative courses were unremarkable.

### Follow-up

The mean follow-up of the study was 1 ± 1 year in the minimally invasive group. Clinical follow-up and echocardiographic parameters were retrieved through records accessed from attendance at the cardiology follow-up clinic.

### Statistical analyses

Statistical analyses were performed with biometrical analysis of sampling software (BIAS 11.06, Epsilon-Verlag, Frankfurt, Germany). Categorical variables are expressed as frequencies. Continuous variables are presented as mean ± standard deviation.

## RESULTS

### Demographic profile

From 2017 to 2020, a total of 27 patients had MVR/mitral valve replacement via a right minithoracotomy as a redo cardiac surgery intervention (minimally invasive group). During the same period, 26 patients underwent resternotomy for mitral valve procedures. The patient preoperative characteristics are presented in Table 1. Forty-eight percent were men. The average EuroSCORE II was 7 ± 3% and the Society of Thoracic Surgeons (STS) score was 11 ± 10% in the minimally invasive group. The STS score in the whole patient cohort was 13 ± 9%. The mean time to reoperation was 7 ± 9 years in the minimally invasive group and 10 ± 13 years totally.

### Previous cardiac surgery

Eighteen patients (66%) had previous MVR (minimally invasive group). The rate of previous aortic valve replacement was 19%. There were 7% with previous CABG in the minimally invasive group. We observed 22% of patients with CAD in the minimally invasive group. However, there was no progression of CAD disease regarding the point in time of reoperation. Therefore no concomitant CABG was necessary.

### Surgical procedure

The operative and perioperative results are listed in Table 2. MVR was performed in 10 patients (37%) in the minimally invasive group. The majority of the patients received mitral valve replacement: 63% in the minimally invasive group and 68% in the whole patient cohort. Conversion to full sternotomy was not necessary in the minimally invasive group. Concomitant tricuspid valve repair was performed in 4% of the minimally invasive group. More concomitant procedures were applied in the resternotomy group (13% tricuspid valve repair, 8% aortic valve replacement). One patient (resternotomy group) who was diagnosed with endocarditis had an additional Bentall procedure due to an aortic root abscess.

**Table 2:** Operative and perioperative results

Operative and perioperative data	Minimally invasive procedure	Resternotomy and minimally invasive procedure
Surgical access, *n* (%)		
Minithoracotomy right	27 (100)	27 (50)
Median resternotomy	0	26 (50)
Mitral valve replacement	17 (63)	36 (68)
Mitral valve repair	10 (37)	17 (32)
Isolated ring implantation	1 (4)	4 (8)
Leaflet plication and ring implantation	6 (22)	7 (13)
Quadrangular excision and ring implantation	1 (4)	1 (2)
Neochord (loop) and ring implantation	2 (7)	3 (6)
Isolated leaflet repair	0	2 (4)
Concomitant procedures		
Tricuspid valve repair, *n* (%)	1 (4)	7 (13)
Tricuspid valve replacement, *n* (%)	0	1 (2)
Aortic valve replacement, *n* (%)	0	4 (8)
Biological conduit (aortic), *n* (%)	0	1 (2)
CPB time (min), mean ± SD	101 ± 39	92 ± 45
CC time (min), mean ± SD	52 ± 26	48 ± 22
ICU stay (days), mean ± SD	3 ± 2	5 ± 4
Rethoracotomy for bleeding, *n* (%)	1 (4)	4 (8)
Blood loss (ml), mean ± SD	566 ± 359	793 ± 410
RBC (unit), mean ± SD	4 ± 4	5 ± 6
In-hospital stay (days), mean ± SD	16 ± 12	18 ± 11
Ventilation time (h), mean ± SD	28 ± 40	39 ± 70
Neurological event (stroke), *n* (%)	0	1 (2)
Wound infection, *n* (%)	1 (4)	2 (4)
Pacemaker implant, *n* (%)	0	1 (2)
LVEF (%), mean ± SD	56 ± 9	52 ± 16
LVEDD (cm), mean ± SD	51 ± 6	48 ± 11
MV max gradient (mmHg), mean ± SD	12 ± 4	13 ± 4
MV mean gradient (mmHg), mean ± SD	4.5 ± 2	4.6 ± 2
30-Day deaths, *n* (%)	1 (4)	6 (11)

CC: cross-clamp; CPB: cardiopulmonary bypass; ICU: intensive care unit; LVEDD: left ventricular end-diastolic diameter; LVEF: left ventricular ejection fraction; MV: mitral valve; RBC: red blood cells; SD: standard deviation.

### Cardiopulmonary results

The cross-clamp time was 52 ± 26 min in the minimally invasive group. We did not observe any vascular complications related to cannulation for CPB.

### Perioperative and postoperative results

The stay in the intensive care unit (ICU) was 3 ± 2 days in the minimally invasive group. The ventilation time was 28 ± 40 min. One patient (4%) in the minimally invasive group had a rethoracotomy because of postoperative bleeding. We observed a blood loss of 566 ± 359 ml in the minimally invasive group. The average transfused red blood cell concentrate was 4 ± 4 units. We observed no neurological events (stroke) in the minimally invasive group and 2% in the whole cohort. Permanent pacemaker implants due to atrioventricular block 3° were not necessary in the minimally invasive group; 2% had the implants in the whole patient cohort. The in-hospital stay was 16 ± 12 days in the minimally invasive group. The 30-day mortality was 4% (*n* = 1) in the minimally invasive group and 11% totally. One patient in the minimally invasive group (with a calculated STS score of 12%) developed right heart failure that required venoarterial extracorporeal membrane oxygenation therapy and died of multiorgan failure on postoperative day 13. Rethoracotomy for reoperation at the mitral valve during the hospital stay was not necessary. No wound infections occurred in our patient cohort. One patient (minimally invasive group) developed a haematoma in the groin that was removed completely. His further postoperative course was unremarkable.

### Postoperative echocardiography

The patients underwent postoperative echocardiography before discharge. We observed a mean left ventricular ejection fraction of 56 ± 9% in the minimally invasive group and 52 ± 16% totally (Table 2). Mitral valve prostheses function was unremarkable in the whole patient cohort; no paravalvular leak was detected. One patient had mild MR after the repair (minimally invasive group).

At the latest follow-up, 80% of the patients in the whole patient cohort were in New York Heart Association (NYHA) functional class I. A total of 20% of the patients in the minimally invasive group were in New York Heart Association functional class II.

Follow-up echocardiography revealed no residual MR greater than grade 1 and no paravalvular leak in the whole patient cohort. The mean mitral valve gradient was 4.5 ± 0.7 mmHg and the mean ejection fraction was 53 ± 10 in the minimally invasive group and similar in the whole patient cohort.

## DISCUSSION

Reoperations in cardiac surgery are reported to be associated with an increased risk for morbidity and mortality [15]. Redo mitral valve surgery is extremely complex technically due to the necessity of re-entering the heart, dense adhesions and difficulties in exposing the valve. Further factors that may explain the increased morbidity and mortality of redo mitral procedures are potential comorbidities of the patients due to previous cardiac surgery, more elderly patients and a prolonged cross-clamp time [16]. A less invasive procedure like transcatheter MVR (e.g. MitraClip) is also an option for MR. However, it is not suitable for all mitral valve pathologies, and the long-term durability of the repair technique has not been investigated sufficiently. Surgical treatment of structural mitral valve pathology is still the gold standard. A great advance in cardiac surgery was achieved when endoscopic minimally invasive mitral valve surgery via a right minithoracotomy was introduced in the 1990s [4]. The feasibility and safety of this technique have been presented in many studies [11–13, 17].

Over the past 10 years, we gained experience in minimally invasive redo mitral valve procedures. The surgical preparation of adhesions around the aorta before placing the Chitwood clamp is tricky and discourages many surgeons from performing redo surgery on the arrested heart. An alternative is the use of the endoclamp technique, which could also lead to potential neurological events due to dislocation of the balloon. In our series, the ascending aorta was mobilized sufficiently and could be clamped with the Chitwood clamp. We have to admit that it is sometimes extremely challenging to deal with the heavy adhesions; therefore, the experience of the surgeon is crucial in this setting. We observed a neurological complication rate of 0% in the minimally invasive redo group (and 2% in the whole patient cohort), which is acceptable. We decided to continue using our clamp technique because of the low neurological complication rate. Other approaches include beating heart mitral valve surgery without clamping the ascending aorta or ventricular fibrillatory arrest. The neurological complication rate could be higher with these methods due to air embolism [18]. However, we do not have experience with these techniques.

The goal of our study was to present the results of patients who underwent minimally invasive redo mitral valve surgery in our centre. We also described retrospectively the whole patient cohort in terms of redo mitral valve procedures (*n* = 53). The patient population in our study was a high-risk group (STS score >10%; EuroSCORE II 7%; respectively 9%; Table 1). We found only 1 paper that included the EuroSCORE among the preoperative characteristics: Hiraoka *et al.* [19] reported a EuroSCORE between 3.8% and 4.8%. Another aspect is the rate of previous cardiac operations. Patients in the minimally invasive group had 66% MVR procedures before and 7% CABG in their anamnesis. Losenno *et al.* [16] described a similar frequency of prior cardiac surgery procedures (CABG and MVR) in his study dealing with minimally invasive redo mitral valve procedures. The operative results were analysed (Table 2). The majority of the patients had a mitral valve replacement (63%). We observed similar results regarding the CPB time (101 ± 39 min in the minimally invasive group) compared to other published data [19]. The ICU stay was 3 ± 2 in the minimally invasive group, which is similar to the results of Hiraoka *et al.* [19] 

(1.8 ± 0.6). The in-hospital stay was in range when compared to the results of Hiraoka *et al.* [19].

The rate of rethoracotomy for bleeding was 4%, which is acceptable for minimally invasive redo procedures. The blood loss was 566 ± 359 ml in the minimally invasive in other published reports. Losenno *et al.* [16] also described reduced transfusion requirements in the minimally invasive group (63% vs 79%; *P* = 0.042) that might be related to the less invasive surgical approach. Bolotin *et al.* [20] made a similar observation (*P* = 0.001). In summary, a minimally invasive approach in redo mitral valve surgery is associated with a low transfusion need and short ICU and ventilation times.

We observed no neurological events (stroke) in the minimally invasive group. However, other published reports include data showing a higher incidence of stroke of up to 5% due to retrograde arterial cannulation and perfusion in minimally invasive procedures [21, 22]. These results do not conform with our observations. The 30-day mortality was 4% in the minimally invasive group and 11% totally. This finding is comparable to those in other published reports (5%) [12, 16, 19]. Other centres report in-hospital mortality rates between 4% and 7% for redo mitral valve procedures [11, 23–25]. The 30-day mortality is acceptable in the high-risk patient cohort that we observed in our study. We did not observe reoperation on the mitral valve in the short-term follow-up period. There were no cases of endocarditis. To summarize, the echocardiographic data in the short-term follow-up period (1 year mean) regarding mitral valve function were good, without MR >1°, and without paravalvular leak or mitral valve stenosis in our cohort. However, a longer follow-up period is necessary to make a statement regarding the durability of the repair technique or mitral valve prosthesis function.

Our results show that a minimally invasive approach for redo mitral valve surgery is safe and feasible. Both surgical techniques (minimally invasive and resternotomy approach) are associated with good postoperative outcomes. However, some of the disadvantages of the minimally invasive technique need to be mentioned. The cannulation of the femoral artery and vein can lead to complications such as retroperitoneal haematoma or dissection of the aorta [26]. We therefore recommend ultrasound and computer tomography monitoring of the femoral artery preoperatively; these modalities help to identify femoral arteries with small diameters, kinking or severe atherosclerosis. Patients with these conditions are not suitable for a minimally invasive approach including peripheral cannulation of the femoral vessels. There are also alternatives like cannulation of the subclavian or carotid artery or the ascending aorta. However, we did not use these cannulation alternatives in our minimally invasive reop mitral surgery series. Even cannulation of both femoral arteries is an interesting strategy in minimally invasive surgery that we did not try before. Another aspect is the difficult de-airing of the heart. Here we recommend transoesophageal guidance and application of carbon dioxide. The minimally invasive approach for a redo mitral valve procedure is a feasible and safe alternative to resternotomy with low blood loss and acceptable duration of ICU stay. However, this technique is challenging. Therefore an experienced surgical team with expertise in minimally invasive techniques is obligatory.

Our results demonstrate a low transfusion requirement and low 30-day mortality in patients undergoing a minimally invasive approach for a redo mitral valve procedure. The video-assisted right anterolateral minithoracotomy for a redo mitral valve operation is safe. However, a randomized controlled trial is necessary to strengthen our findings.

There are selection biases in minimally invasive versus resternotomy subgroups in our series. Among the preoperative patient characteristics (Table 1), some differences between the 2 subgroups stand out. In the resternotomy group, we observed more patients with endocarditis (15% vs 4%), CAD (46% vs 22%), chronic renal failure (35% vs 22%) and peripheral artery disease. These parameters lead to higher calculated EuroSCOREs and STS scores in the resternotomy group. There were also more concomitant procedures involving the aortic valve and tricuspid valve performed in the resternotomy group (Table 2). Therefore the resternotomy group had a higher operative risk that reflects the inferior outcome of this subgroup regarding 30-day mortality (19% vs 4%). For a better comparison of the 2 subgroups, a propensity matched analysis is necessary. We illustrated the results from the minimally invasive group and the whole group (minimally invasive and resternotomy together) without a statistical comparison.

### Limitations

Our study was retrospective in nature with only a short follow-up period. The durability of the repair has to be confirmed over a longer period. A small sample size and the lack of a propensity matched cohort are additional limitations of our study.

**Conflict of interest:** none declared.

## ABBREVIATIONS

CABG: Coronary artery bypass grafting
CAD: Coronary artery disease
CPB: Cardiopulmonary bypass
ICU: Intensive care unit
MR: Mitral valve regurgitation
MVR: Mitral valve repair
NYHA: New York Heart Association
STS: Society of Thoracic Surgeons
